# Combined effect of hydrogen sulphide donor and losartan in experimental diabetic nephropathy in rats

**DOI:** 10.1186/s40200-015-0185-7

**Published:** 2015-07-28

**Authors:** Manpreet Kaur, Shilpi Sachdeva, Onkar Bedi, Tavleen Kaur, Puneet Kumar

**Affiliations:** Pharmacology Division, Department of Pharmacology, ISF College of Pharmacy, Moga, 14200 Punjab India

**Keywords:** Diabetes mellitus, Diabetic nephropathy, Streptozotocin, Sodium hydrosulphide

## Abstract

**Background:**

Diabetic nephropathy (DN) is one of the complex complications of Diabetes Mellitus (DM). The present study has been designed to examine protective role of hydrogen Sulphide (H_2_S) donor against streptozotocin (STZ) -induced behavioral, oxidative abnormalities and its DN like symptoms in rats.

**Methods:**

For the induction of DN single intraperitoneal administration of STZ (45 mg/kg) was given till third week. Behavioral parameters were measured on 1st, 7th, 21st and 42nd days and biochemical parameters were performed on 42nd day. All the drug treatments [NaHS (10 & 30 μmol/kg i.p), DL-propargylglycine (10 mg/kg i.p), standard drug- Losartan (5 mg/kg p.o)] were given for 3 weeks staring from 21st day after the STZ injection.

**Results:**

Three weeks treatment with sodium hydrosulphide (NaHS) (10 and 30 μmol/kg i.p,) significantly attenuated the behavioral and biochemical abnormalities in STZ-treated animals. DL-propargylglycine (10 mg/kg i.p) pretreatment with sub-effective dose of NaHS (30 μmol/kg i.p) significantly reversed the protective effect of NaHS. However, combination of both NaHS (30 μmol/kg i.p) and standard drug losartan (5 mg/kg p.o) potentiated their effects as compared to their effect alone.

**Conclusion:**

The results of the present study suggest that H_2_S treatment showed significant improvement in behavioral and biochemical abnormalities induced by STZ administration. Thus H_2_S represents a target of treatment to prevent the progression of complications by DN.

## Background

Diabetic nephropathy (DN) is the most common and serious complication of DM, which affects a large population worldwide [1, 2]. However, the pathogenesis involved in DN is still not clear [1–3]. DN is defined as partial loss of function of kidney associated with nephrotic syndrome is characterized by a glomerulosclerosis, glomerular and renal-cell hypertrophy, podocyte loss, reduction in GFR, elevation of MABP and fluid retention [4].

The single dose administration of STZ (45 mg/kg i.p.) in rats has been shown to produce DN after 3–6 weeks. Generally, three days (or) one week after STZ, animal should be screened and those with fasting blood glucose above 240 mg/dl are generally included in the studies of DN [5]. Now days, various drug strategies are being used to understand the pathogenesis of DN and to design suitable drug strategies.

Hydrogen sulfide (H_2_S) is a third gaseous bioactive substance produced in different mammalian cells, both exogenous and endogenous H_2_S have been reported to cause vascular smooth muscle relaxation and decrease in blood pressure [6], thus a physiological vasodilator [7]. H_2_S significantly participates in the control of renal functions and renal protection [8], including glomerular and tubular functions, hypertension, atherosclerosis and cardiac/renal ischemia–reperfusion injuries [9]. Also it has been reported that H_2_S play important roles in cell proliferation and apoptosis [10], neurotransmission, inflammatory processes [11–13], cirrhosis, sepsis, neurodegenerative disease, erectile dysfunction, and asthma [14].

Currently available treatments *i.e.*, both ACE inhibitors and AT-I receptor antagonists are associated in compensatory decrease in release of renin. This may compromise the long term beneficial effects of these drugs. However, ACE inhibitors may often associate with dry cough, angioedema as side effect. In this regard H_2_S donor is being suggested as better alternatives to ACE inhibitors. H_2_S is shown to decrease the synthesis and release of renin may produce additional beneficial effects. However, to our knowledge there is no previous work has been carried out to explore the protective effect of NaHS against STZ-induced DN in rats.

Therefore, present study has been designed to elucidate the effect of NaHS and its possible renin mechanism against STZ-induced behavioral, biochemical and histological abnormalities.

## Methods

### Animals

Male Wistar rats (250–300 g) bred in the Central Animal House facility of I.S.F. College of Pharmacy, Moga, and Punjab, India were used in the present study. Animals were acclimatized to laboratory conditions prior to experiments. They were housed in-group of three, under standard laboratory conditions of temperature (22 ± 1 °C), relative humidity (60 %) and light/dark cycle. Rats were fed on standard chow diet and water *ad libitum*. The experimental protocol was approved by the Institutional Animal Ethics Committee (IAEC) of ISF College of Pharmacy, Moga, Punjab, approval no. 162 dated 08 march 2014 and was carried out in accordance with the guidelines of the Indian National Science Academy (INSA) for the use and care of experimental animals.

### Drugs and chemicals

Sodium Hydrosulphide and DL-propargylglycine were purchased from Sigma Aldrich Ltd, (St. Louis, USA). Losartan received as a gift sample from Kwality Pharmaceuticals Pvt. Ltd. (Amritsar). All other chemicals and reagents used in present study were of analytical grade and freshly prepared.

### Treatment schedule

Total forty-two rats of either sex were used in study. The animals were randomly divided into seven experimental groups consisting of 6 animals in each (n =6) as given in Table 1.

**Table 1:** Showing experimental grouping and doses used

Group	Treatment
1	Normal control
2	Disease control (STZ treated 45 mg/kg, i.p.)
3	STZ+ NaHS (10 μmol/kg i.p)
4	STZ+ NaHS (30 μmol/kg i.p)
5	STZ+ DL-Propergylglycine (10 mg/kg i.p) + NaHS (30 μmol/kg i.p)
6	STZ+ Losartan (5 mg/kg p.o)
7	STZ+ Losartan (5 mg/kg p.o) + NaHS (10 μmol/kg i.p)

**Fig. 1:**
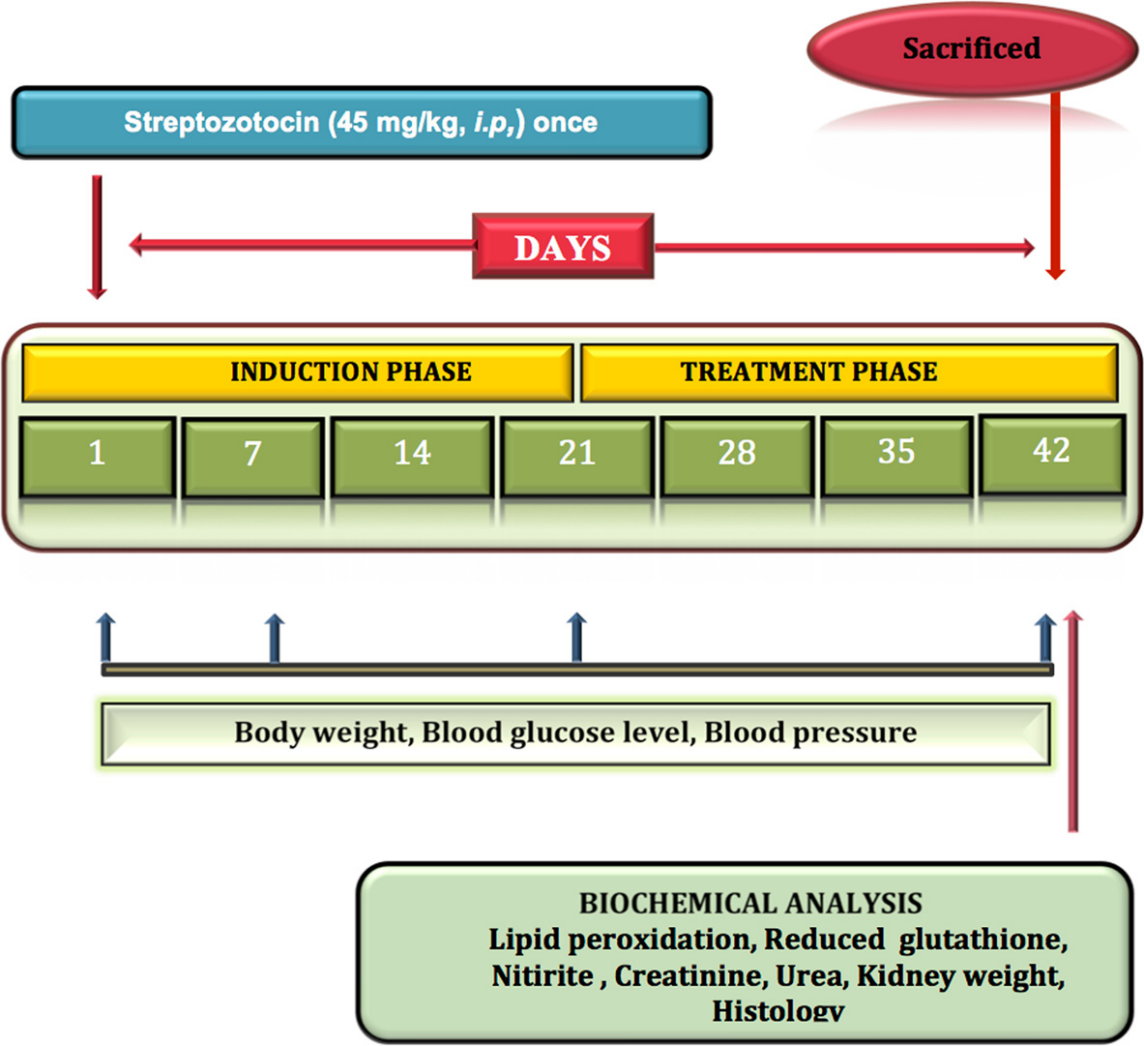
Effect of NaHS on blood glucose level in STZ treated rats. ^a^P < 0.05 *versus* vehicle treated, ^b^P < 0.05 *versus* [STZ (45)] treated group, ^c^P < 0.05 *versus* [STZ (45) + NaHS (10)] treated group, ^d^P < 0.05 *versus* [STZ (45) + NaHS (30)] treated group. STZ = Streptozotocin, NaHS = Sodium hydrosulphide, LOS = Losartan, DL-p = DL-propargylglycine

All the drugs in the present study were given for three weeks staring from 21st day after the STZ induced DN. The body weight, blood glucose level and blood pressure were measured on 1st, 7th, 21st, 42nd days after STZ administration. Terminally on 6th week animals were sacrificed and kidneys were isolated to assess kidneys weight and markers of oxidative stress such as reduced glutathione, lipid peroxidation, and nitrite levels. The serum urea and creatinine levels were also measured by using kits on 42nd day. The Histopathological studies were carried out for different groups to estimate the pathological changes of kidney. The experimental procedure is summarized in Fig. 1.

### Induction of experimental diabetic nephropathy

Diabetes mellitus was induced by single injection of STZ (50 mg/kg, i.p.) (Vaishya *et al.*, 2008), dissolved in freshly prepared ice-cold citrate buffer (pH 4.5). After 1 week of STZ administration animals having random serum glucose more than 240 mg/dl were considered as diabetic. The nephropathy developed after 3 weeks of STZ administration.

### Assessment of STZ-induced diabetes

#### Estimation of serum glucose

At the end of the experimental protocol, the blood samples were collected from retro-orbital sinus and the serum was separated. The glucose concentration was estimated by glucose oxidase-peroxidase (GOD-POD) method using commercially available kits (Coral Clinical System, Goa, India) [15].

#### Estimation of body weight

Body weight was estimated in grams on day 1st, 7th, 21st, 42nd.

### Assessment of DN

#### Estimation of serum creatinine (SC)

The serum creatinine concentration was estimated on 42nd day by alkaline picrate method using commercially available kit (Coral system, Goa, India) [16].

#### Estimation of blood urea nitrogen (BUN)

The BUN was estimated at day 42th by Berthelot method using the commercially available kit (Coral clinical system, Goa, India [17].

#### Estimation of kidney weight/body weight (%)

Both left and right kidneys were isolated at the end of study on 42th day, renal fascia was removed and kidneys were weighed individually. Total kidney weight/body weight (%) was calculated according to following formula [18, 19].

$$ \mathrm{Calculation} = \frac{\ \mathrm{Left}\ \mathrm{kidney}\ \mathrm{weight}\ \left(\mathrm{g}\mathrm{m}.\right) + \mathrm{Right}\ \mathrm{kidney}\ \mathrm{weight}\ \left(\mathrm{g}\mathrm{m}.\right)\ }{\mathrm{Body}\ \mathrm{weight}\ \left(\mathrm{g}\mathrm{m}.\right)}\kern0.75em  \times 100 $$

#### Assessment of renal oxidative stress

The development of oxidative stress in the kidney assessed by estimating renal thiobarbituric acid reactive substance (TBARS), reduced form of glutathione (GSH) and nitrite.

#### Tissue preparation

Animals were sacrificed on day 15 by decapitation and the kidneys were removed and rinsed with ice-cold isotonic saline. Liver separated out and weighed. Kidney samples were then homogenized with ice-cold 0.1 mol/L phosphate buffer (pH 7.4) 10 times (w/v). The homogenate was centrifuged at 10,000 g for 15 min and aliquots of supernatant were separated and used for biochemical estimation.

#### Measurement of lipid peroxidation

The quantitative measurement of lipid peroxidation in kidney was performed according to the method of Will’s, 1965. The amount of malondialdehyde (MDA), a measure of lipid peroxidation was assayed in the form of thiobarbituric acid reacting substances (TBARS). TBARS were quantified using an extinction coefficient of 1.56 × 105 M^−1^ cm^−1^ and expressed as nmol of MDA per mg protein [20].

#### Estimation of reduced glutathione

Reduced glutathione was estimated according to the method described by Ellman, 1959. Reduced Glutathione levels were measured at 412 nm using a Perkin Elmer Lambda 20 spectrophotometer were calculated using molar extinction co-efficient of the chromophore (1.36 × 104 (mol/L) ^−1^ cm^−1^) [21].

#### Estimation of nitrite

The accumulation of nitrite in the supernatant, an indicator of the production of nitric oxide (NO), was determined with a colorimetric assay with Greiss reagent [0.1 % N- (1-naphthyl) ethylenediame dihydrochloride, 1 % sulfanilamide and 2.5 % phosphoric acid] as described by Green [22].

#### Estimation of protein

Protein estimation was done by using protein estimation kit (Coral kit).

### Measurement of mean arterial blood pressure (MABP)

The mean arterial blood pressure was recorded in rats by tail-cuff apparatus (NIBPMP100, Biopac) containing sensitive photoelectric sensors. To create sufficiently large pulse volume oscillations, rats were exposed to heat for about 12 min at 38 ° C prior to recording the pressure. The heating increased the mean arterial pressure by an average of 4 ± 2 mmHg, as indicated by direct measurement of pressure. Three different sizes of cuffs were tested. The MAP determined at maximum pulse volume oscillations coincides fairly well with the true mean arterial pressure [23].

### Histological studies

The early changes in glomeruli were assessed histologically as previously described [24]. The kidney was excised and immediately immersed in 10 % formalin. The kidney was dehydrated in graded concentrations of alcohol, immersed in xylene and then embedded in paraffin. From the paraffin blocks, sections of 3 μm in thickness were made and stained with Hematoxylin and Eosin (H & E) to assess the pathological changes occurs in glomeruli using light microscopy (10×).

### Statistical analysis

Values are expressed as mean ± SEM. The data for behavioral analysis were statistically analysed using two-way ANOVA followed by Bonferroni post hoc test were employed. The data for biochemical parameters were analysed using two-way ANOVA test. A value of p < 0.05 was considered to be statistically significant.

## Results

### Assessment of diabetes mellitus

#### Effect of NaHS on blood glucose level in STZ treated rats

The blood glucose levels were increased markedly in STZ treated rats on 7th, 21st, 42nd day as compared to control group. Treatment with NaHS (10 & 30 μmol/kg i.p., 21 days) significantly decreased blood glucose level in STZ treated rats as compared to vehicle treated group. The losartan (5 mg/kg) alone had no effect on blood glucose levels. Further, pretreatment with DL-p (H_2_S inhibitor) was given along with NaHS (30 μmol/kg), it significantly reversed the protective effect of NaHS (30 μmol/kg) as compared with its effect alone. The combination of losartan (5 mg/kg) and NaHS (10 μmol/kg) also not produced synergistic effect on blood glucose levels in STZ treated rats Fig. 1.

#### Effect of NaHS and losartan on body weight in STZ treated rats

STZ treated rats shows significant decrease (~110 g) in body weight on day 7th, 21st, 42nd as compared to control group (~290 g). Treatment with NaHS (10 & 30 μmol/kg i.p., 21 days) and losartan (5 mg/kg) significantly increased body weight (~168 g, ~190 g respectively) in STZ treated rats as compared to vehicle treated group. Further, pretreatment with DL-p (H_2_S inhibitor) was given along with NaHS (30 μmol/kg), it significantly reversed (~148 g) the protective effect of NaHS (30 μmol/kg) as compared with its effect alone. The combination of NaHS (10 μmol/kg) and losartan (5 mg/kg) produced synergistic effect (~215 g) as compared to their effects alone (~168 g, 165 g respectively) in STZ treated rats.

### Assessment of DN

#### Effect of NaHS and losartan on serum creatinine (SC) levels in STZ treated rats

The SC levels were noted to be increased (1.8 mg/dl) markedly in STZ treated rats on day 42nd as compared to control group (0.8 mg/dl). Treatment with NaHS (10 & 30 μmol/kg i.p., 21 days) and losartan (5 mg/kg) significantly decrease SC levels (1.63 mg/dl, 1.45 mg/dl, 1.50 mg/dl respectively) in STZ treated rats as compared to vehicle treated group. Further, pretreatment with DL-p (H_2_S inhibitor) was given along with NaHS (30 μmol/kg), it significantly reversed (1.68 mg/dl) the protective effect of NaHS (30 μmol/kg) as compared with its effect alone. The combination of NaHS (10 μmol/kg) and losartan (5 mg/kg) shows synergistic effect (1.18 mg/dl) in SC levels as compared to their effects alone (1.63 mg/dl, 1.57 mg/dl) in STZ treated rats.

#### Effect of NaHS and losartan on blood urea nitrogen levels (BUN) in STZ treated rats

STZ treated rats shows significant increase (75 mg/dl) in blood urea nitrogen levels on day 42nd as compared to control group (25 mg/dl). Treatment with NaHS (10 & 30 μmol/kg i.p., 21 days) and losartan (5 mg/kg) significantly decreases BUN levels (65, 52, 66 mg/dl respectively) in STZ treated rats as compared to vehicle treated group. Further pretreatment with DL-p (H_2_S inhibitor) was given along with NaHS (30 μmol/kg), it significantly reversed (68 mg/dl) the protective effect of NaHS (30 μmol/kg) as compared with its effect alone. The combination of NaHS (10 μmol/kg) and losartan (5 mg/kg) shows synergistic effect (37 mg/dl) in BUN levels as compared to their effects alone in STZ treated rats.

#### Effect of NaHS and losartan on kidney weight/body weight (KW/BW) ratio in STZ treated rats

STZ treated rats shows significant decrease in KW/BW ratio (1.9 %) on day 42nd as compared to control group (3.83 %). Treatment with NaHS (10 & 30 μmol/kg i.p., 21 days) and losartan (5 mg/kg) significantly increase KW/BW ratio (2.43, 2.94, 3.5 % respectively) in STZ treated rats as compared to vehicle treated group. Further, pretreatment with DL-p (H_2_S inhibitor) was given along with NaHS (30 μmol/kg), it significantly reversed (2.2 %) the protective effect of NaHS (30 μmol/kg) as compared with its effect alone. Whereas, the combination of NaHS (10 μmol/kg) and losartan (5 mg/kg) produced synergistic effect (3.4 %) in KW/BW ratio as compared to their effects alone in STZ treated rats.

### Assessment of oxidative stress

#### Effect of NaHS and losartan on lipid peroxidation (LPO) levels in STZ treated rat

**Fig. 2:**
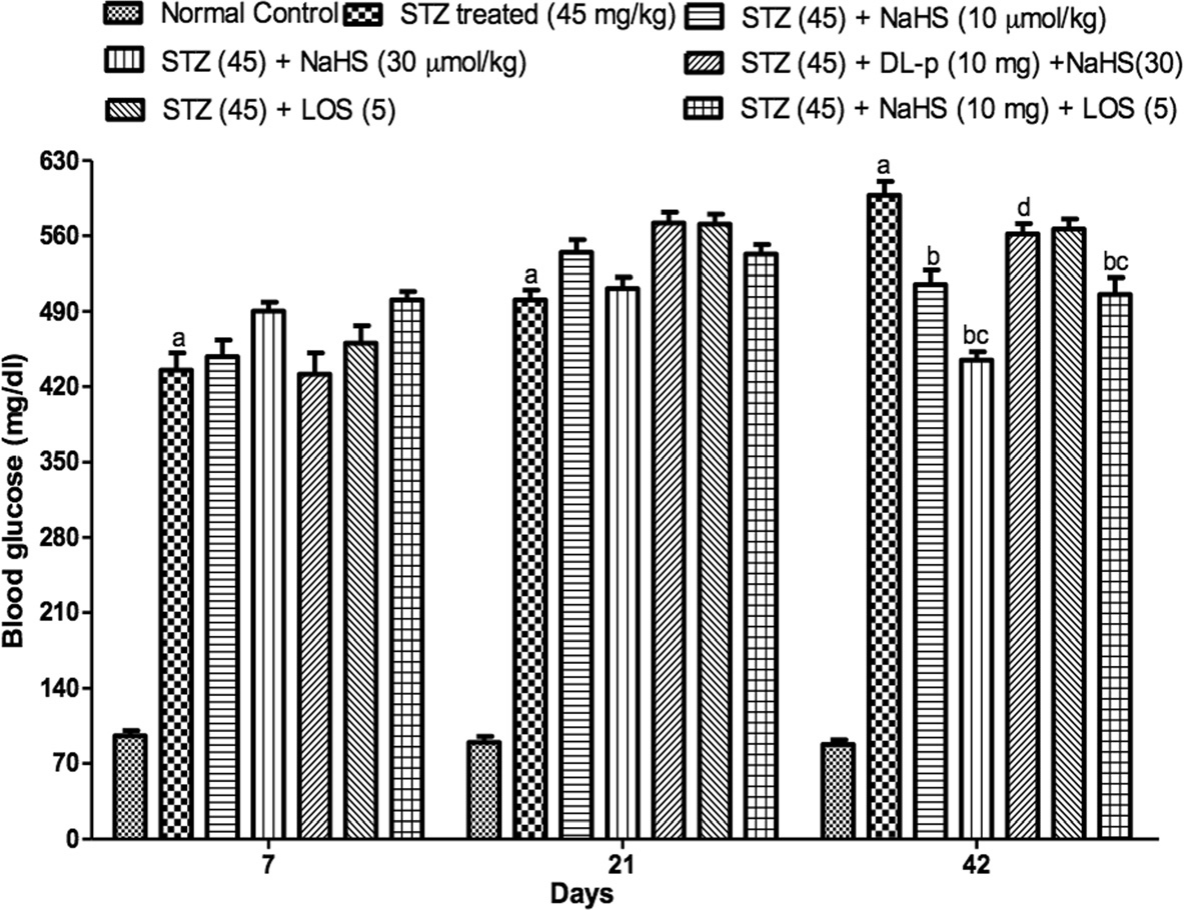
Effect of NaHS and losartan on Lipid Peroxidation in STZ treated rats. ^a^P < 0.05 *versus* vehicle treated, ^b^P < 0.05 *versus* [STZ (45)] treated group, ^c^P < 0.05 *versus* [STZ (45) + NaHS (10)] treated group, ^d^P < 0.05 *versus* [STZ (45) + NaHS (30)] treated group, ^e^P < 0.05 *versus* [STZ (45) + LOS (5)] treated group. STZ = Streptozotocin, NaHS = Sodium hydrosulphide, LOS = Losartan, DL-p = DL-propargylglycine

STZ treated rats shows significant increase in LPO levels on day 42nd as compared to control group. Treatment with NaHS (10 & 30 μmol/kg i.p., 21 days) and losartan (5 mg/kg) significantly decrease in LPO levels in STZ treated rats as compared to vehicle treated group. Further, pretreatment with DL-p (H_2_S inhibitor) was given along with NaHS (30 μmol/kg), it significantly reversed the protective effect of NaHS (30 μmol/kg) as compared with its effect alone. Whereas, the combination of NaHS (10 μmol/kg) and losartan (5 mg/kg) produced synergistic effect in LPO as compared to their effects alone in STZ treated rats Fig. 2.

#### Effect of NaHS and losartan on reduced glutathione levels

**Fig. 3:**
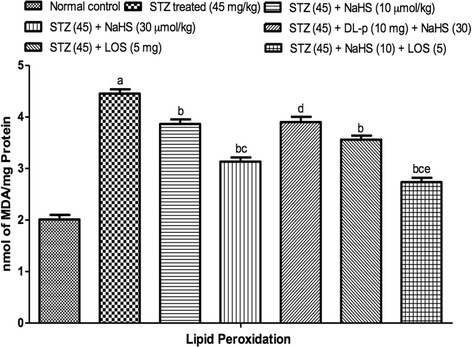
Effect of NaHS and losartan on Reduced Glutathione in STZ treated rats. ^a^P < 0.05 *versus* vehicle treated, ^b^P < 0.05 *versus* [STZ (45)] treated group, ^c^P < 0.05 *versus* [STZ (45) + NaHS (10)] treated group, ^d^P < 0.05 *versus* [STZ (45) + NaHS (30)] treated group, ^e^P < 0.05 *versus* [STZ (45) + LOS (5)] treated group. STZ = Streptozotocin, NaHS = Sodium hydrosulphide, LOS = Losartan, DL-p = DL-propargylglycine

STZ treated rats shows significant decrease in GSH levels on day 42nd as compared to control group. Treatment with NaHS (10 & 30 μmol/kg i.p., 21 days) and losartan (5 mg/kg) significantly increase in GSH levels in STZ treated rats as compared to vehicle treated group. Further, pretreatment with DL-p (H_2_S inhibitor) was given along with NaHS (30 μmol/kg), it significantly reversed the protective effect of NaHS (30 μmol/kg) as compared with its effect alone. Whereas, the combination of NaHS (10 μmol/kg) and losartan (5 mg/kg) produced synergistic effect in LPO as compared to their effects alone in STZ treated rats Fig. 3.

#### Effect of NaHS and losartan on nitrite levels in STZ treated rats

**Fig. 4:**
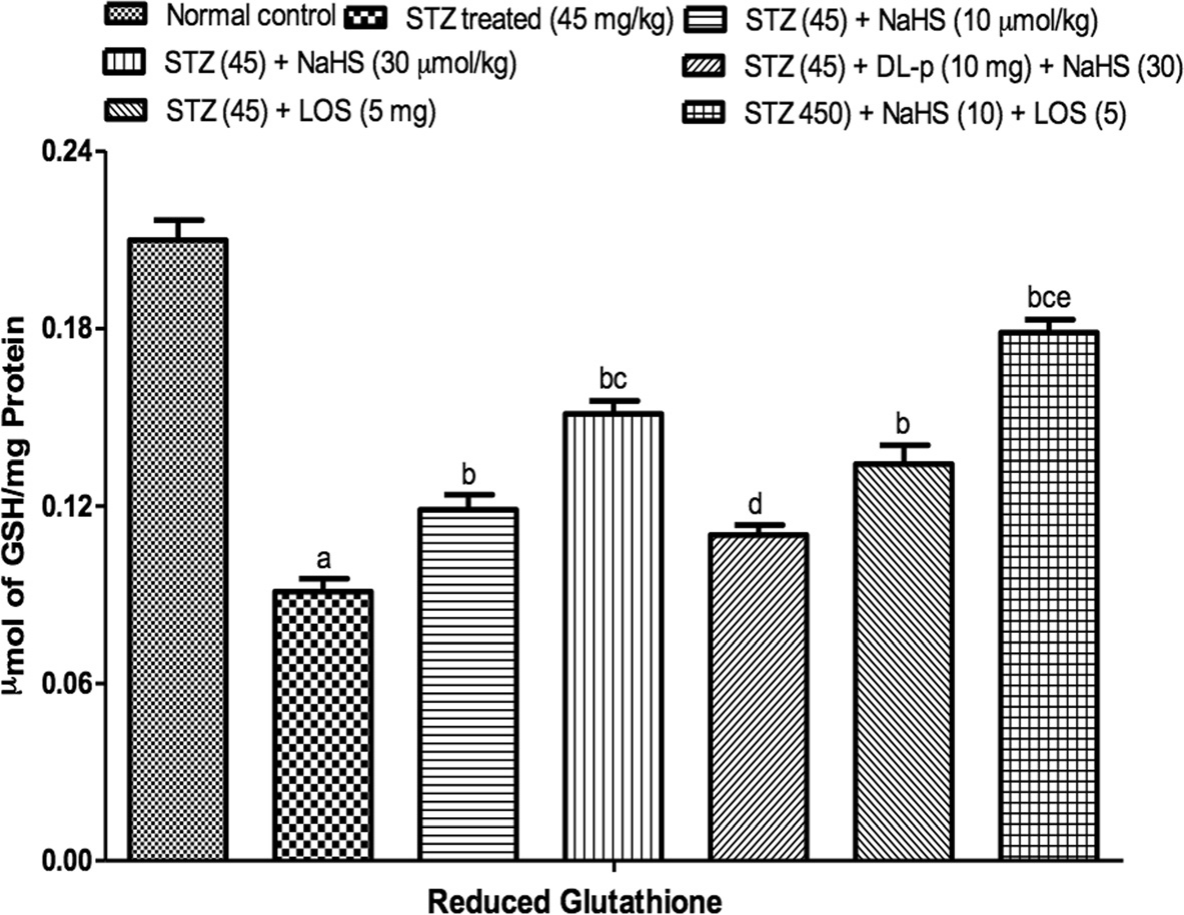
Effect of NaHS and losartan on Nitrite in STZ treated rats. ^a^P < 0.05 *versus* vehicle treated, ^b^P < 0.05 *versus* [STZ (45)] treated group, ^c^P < 0.05 *versus* [STZ (45) + NaHS (10)] treated group, ^d^P < 0.05 *versus* [STZ (45) + NaHS (30)] treated group, ^e^P < 0.05 *versus* [STZ (45) + LOS (5)] treated group. STZ = Streptozotocin, NaHS = Sodium hydrosulphide, LOS = Losartan, DL-p = DL-propargylglycine

STZ treated rats shows significant increase in nitrite levels on day 42nd as compared to control group. Treatment with NaHS (10 & 30 μmol/kg i.p., 21 days) significantly decrease in nitrite levels in STZ treated rats as compared to vehicle treated group. Further, pretreatment with DL-p (H_2_S inhibitor) was given along with NaHS (30 μmol/kg), it significantly reversed the protective effect of NaHS (30 μmol/kg) as compared with its effect alone. The losartan (standard drug) also shows significant decrease in nitrite levels in STZ treated rats. Whereas, the combination of NaHS (10 μmol/kg) and losartan (5 mg/kg) produced synergistic effect in nitrite as compared to their effects alone in STZ treated rats Fig. 4.

### Effect of NaHS and losartan on mean arterial blood pressure (MABP)

**Fig. 5:**
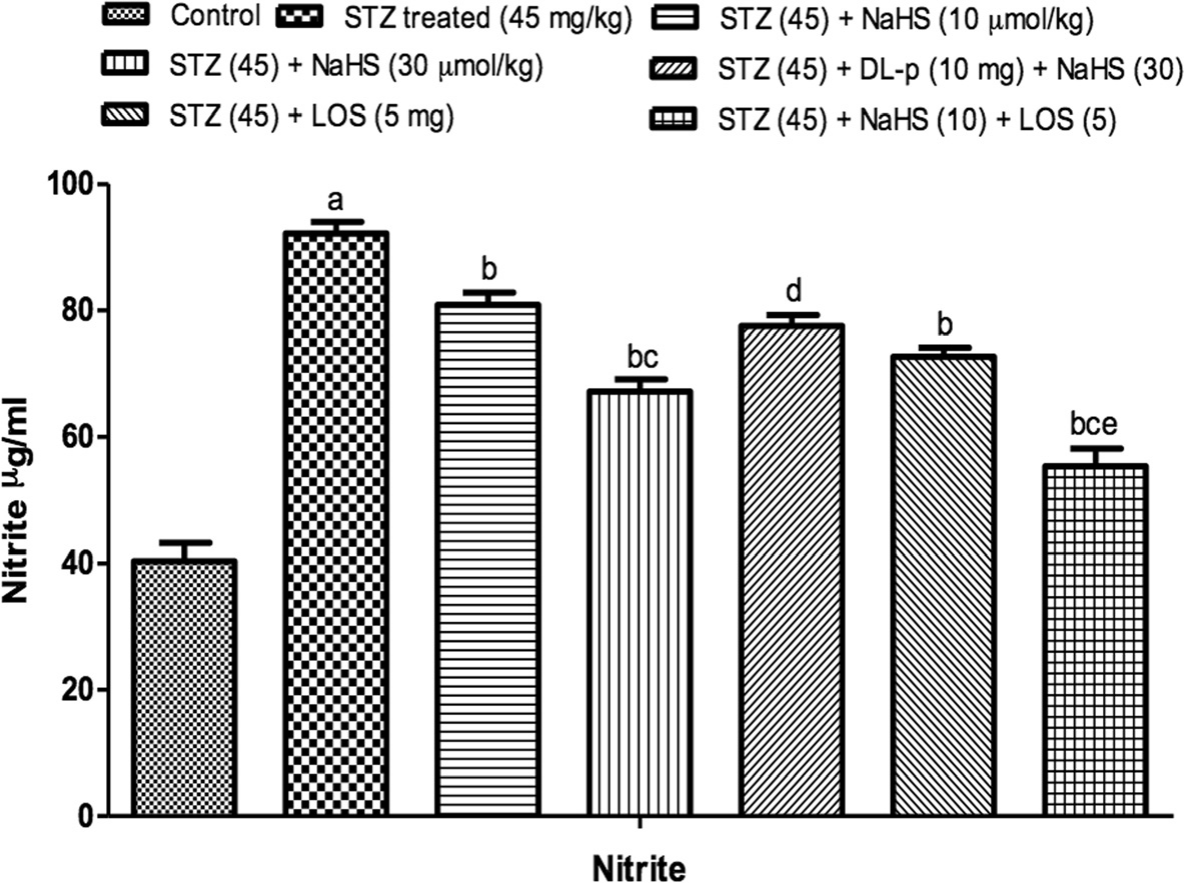
Effect of NaHS and losartan on MABP in STZ treated rats. ^a^P < 0.05 *versus* vehicle treated, ^b^P < 0.05 *versus* [STZ (45)] treated group, ^c^P < 0.05 *versus* [STZ (45) + NaHS (10)] treated group, ^d^P < 0.05 *versus* [STZ (45) + NaHS (30)] treated group, ^e^P < 0.05 *versus* [STZ (45) + LOS (5)] treated group. STZ = Streptozotocin, NaHS = Sodium hydrosulphide, LOS = Losartan, DL-p = DL-propargylglycine

STZ treated rats shows significant increase in MABP levels on day 7th, 21st, 42nd as compared to control group. Treatment with NaHS (10 & 30 μmol/kg i.p., 21 days) and losartan (5 mg/kg) significantly decrease in MABP in STZ treated rats as compared to vehicle treated group. Further, pretreatment with DL-p (H_2_S inhibitor) was given along with NaHS (30 μmol/kg), it significantly reversed the protective effect of NaHS (30 μmol/kg) as compared with its effect alone. Whereas, the combination of NaHS (10 μmol) and losartan (5 mg) produced synergistic effect in MABP levels as compared to their effects alone in STZ treated rats Fig. 5.

### Effect of NaHS and losartan on on renal histological study

**Fig. 6:**
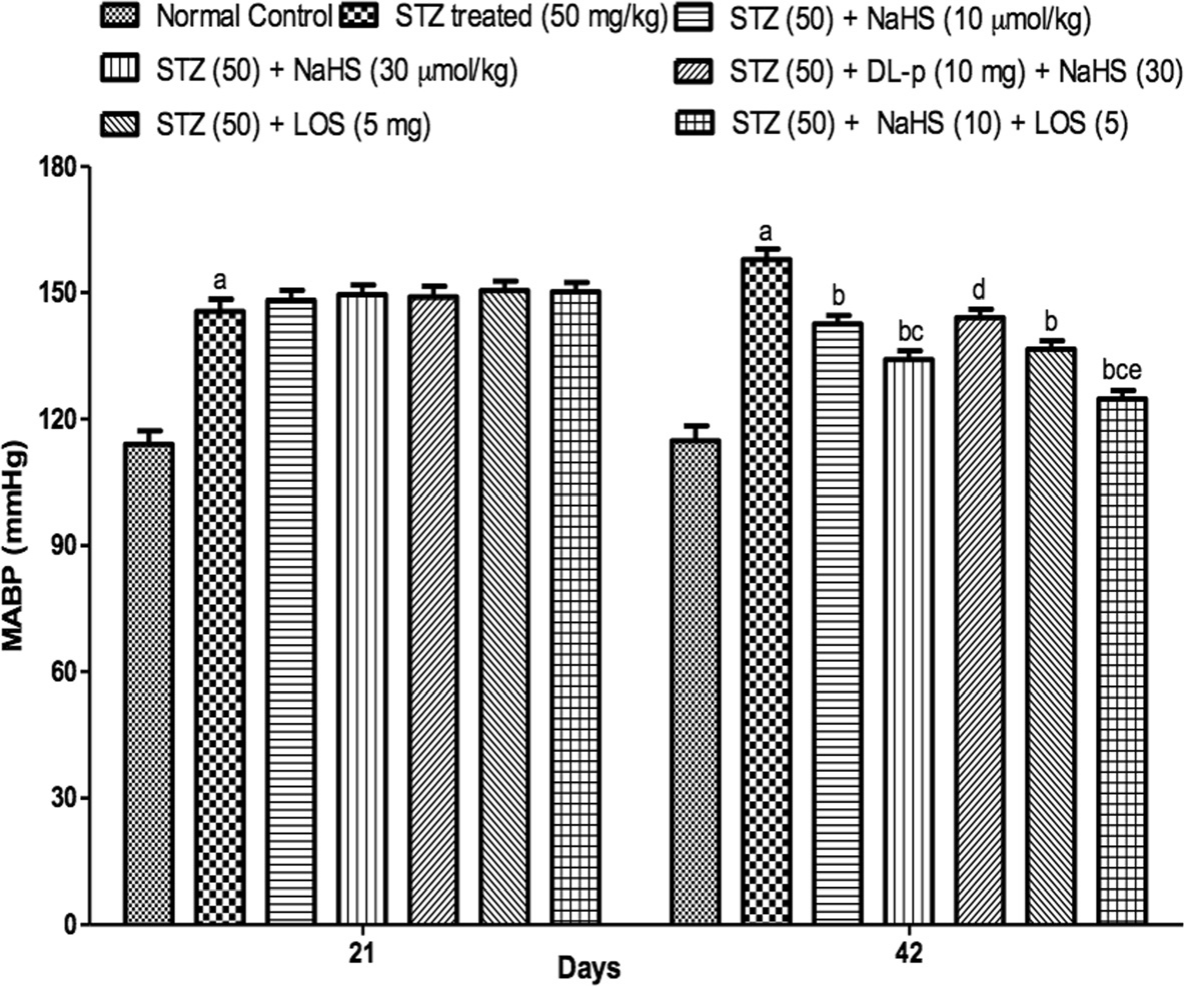
Hemotoxylin-Eosin stained longitudinal section of kidneys (10×). A- Normal control, B- STZ treated group (45), C- NaHS (30), D- DL-p (10) + NaHS (30), E-LOS, F- NaHS (10) + Losartan (5)

The histopathological study shows that STZ administration results necrosis in glomerulus. NaHS and losartan alone produced significant effects. Further treatment with combinations of NaHS (10) + losartan (5) significantly recovered histopathological changes. Whereas pretreatment with inhibitor reverse the beneficial effects produced by NaHS (30) Fig. 6.

## Discussion

Diabetic Nephropathy was produced with single dose administration of STZ (45 mg/kg i.p.) in rats. STZ-induced DM rodents show secondary complications after 3–6 weeks, as assessed in terms of SC, BUN, proteinuria, CC, which is often associated with extracellular matrix deposition, dyslipidemia and development of glomerulosclerosis and tubulointerstitial fibrosis [25]. Generally, 3 days (or) 1 week after STZ, animal should be screened and those with fasting blood glucose above 240 mg/dL are generally included in the studies of DN [26]. STZ damages the DNA of the pancreatic-β cells and triggers multiple pathways, including activation of protein kinase-C, poly (ADP-ribose) polymerase and NAD(P)H oxidase, with consequent generation of ROS and advanced glycation end products resulting in renal damage and nephropathy [25–27]. The experimental evidence suggests that STZ-induced diabetes downregulates the expression and activity of eNOS and decreases the bioavailability of NO, which aggravates nephropathy [5].

STZ treated rats shows significant increase in blood glucose levels by increase the formation ROS by breaking the single strand of DNA which leads to the activation of PARP and result in apoptotic and necrotic death of Islets of Langerhans [28, 29]. Nowadays, accumulating evidences show that abnormal insulin regulation secretion has been proved to be a significant effect on the weight loss of DN patients. But the treatment with NaHS at low and high dose significantly reduced the levels of blood glucose levels by protecting the β-cells form the STZ induced damage to β-cells and it also promote the secretion of insulin [30]. Whereas losartan did not produce any significant effect on blood glucose levels.

One of the most sensitive and dramatic indicators of kidney injury is to increase the creatinine and urea level in serum and in hepatocyte injury is the release of intracellular enzymes, such as transaminases and serum alkaline phosphatase in the circulation after STZ administration [31, 32]. In addition, the soluble enzymes ALT/AST are released when injury involves organelles such as mitochondria [33]. Elevation of these levels causes severe damage to nephron, which indicates the abnormal kidney functioning, which was considered as significant markers of renal dysfunction [34]. After the 3-week treatment with NaHS and losartan alone or in combination produce significantly decrease in serum creatinine and BUN levels in STZ-diabetic rats [35].

The incidence and severity of lesions produced by STZ in pancreas, liver, kidney and GIT, progressively increased with time from one to six weeks post treatment, which results increase in kidney weight/body weight ratio as compared with the control group [36]. But treatment with NaHS and losartan alone or in combination showed a significant improvement in reduced body weight and increased kidney weight/body weight ratio.

A growing body of evidence showed that oxidative stress (OS) played a crucial role in the development and progression of DN [37]. ROS, the result of excessive production of reactive oxygen species (ROS) [38], can induce mitochondrial dysfunction, decline adenosine triphosphate, and then lead to DN [39]. Advanced glycation end products (AGEs), playing a central role in DN, are accumulated in glomerular basement membrane, mesangial cells, and endothelial cells [40]. The interaction between AGEs and their specific receptors could trigger OS damage and then lead to signaling cascade events [37]. Transforming growth factor beta family (TGF-β) was reported to have great relationships with OS *via* the regulation of protein levels of antioxidant enzymes such as superoxide dismutase (SOD), glutathione peroxidase (GSH-PX), catalase (CAT), and others [41]. The present study show that there is significant (p < 0.05) decrease in the LPO, nitrite and significant (p < 0.05) increase in the level of GSH in STZ treated group so which revealed the antioxidant action of NaHS and losartan.

STZ administration (45 mg/kg, *i.p,* once) resulted significant increase in MABP. From the previous studies it has been observed that the development of renovascular hypertension depends on the release of renin from the juxtaglomerular (JG) cells, a process regulated by intracellular cAMP. Hydrogen sulfide (H_2_S) downregulates cAMP production in some cell types by inhibiting adenylyl cyclase, suggesting the possibility that it may modulate renin release. Here, we investigated the effect of H_2_S on plasma renin activity and BP in rat models of STZ induced DN hypertension.

The H_2_S is beneficial at physiological concentrations but detrimental at supra physiological concentrations. H_2_S significantly participates in the control of renal functions, including glomerular and tubular functions.

We postulated that hyperglycemia would also decrease CSE expression in the kidney, which may cause renal microcirculation injury and renal ischemia [42, 43]. Endothelial dysfunction attributable to eNOS reduction and insulin deficiency might induce decreased peritubular capillaries blood flow, resulting in tubulointerstitial ischemia and injury [44]. Anti-apoptotic effects of H_2_S have also been reported in other types of cells [45]. H_2_S also promotes angiogenesis through VEGF signaling pathways such as the PI3K-Akt pathway [45]. As the biological features of H_2_S resemble those of NO, modulation of H_2_S production might be involved in diabetic tubulointerstitial ischemia. Further, high glucose further induces the CSE expression in the β-cells in pancreas, in contrast to the renal proximal tubules [46]. These suggest that H_2_S may protect β-cells from glucotoxicity, eventually leading to the promotion of insulin secretion [47]. It has been demonstrated that NaHS administration increased blood flow by PTC dilation. Indeed, CSE reduction results in decreased H_2_S formation [47].

Cytoprotection by H_2_S is associated with the inhibition of reactive oxygen species (ROS) production *via* inhibition of NADH oxidase [48], the induction of anti-oxidative molecules such as thioredoxin [49], and an increase in glutathione (GSH) production [50]. Therefore, β-cell protection is the focus of new strategies for the treatment of diabetes [30]. At physiological concentrations of H_2_S produce anti-inflammatory effects [14]. Similarly, H_2_S inhibits endotoxin induced up regulation of iNOS expression, NO production and tumor necrosis factor-a (TNF-a) expression in cultured microglia [12].

In conclusion, these results demonstrate that H_2_S may inhibit renin activity by decreasing the synthesis and release of renin, suggesting its potential therapeutic value for renovascular hypertension.

## Conclusions

In conclusion, we hypothesized NaHS showed their protective effect by increasing the production of H_2_S by the activation of CSE enzyme that ultimately leads to minimizing the secondary complication of the DM in STZ induced DN. Further hydrogen sulphide donor produce synergistic effect along with standard Losartan. Therefore we conclude that NaHS has shown protective effect in DN rats but combined treatment with standard produce more significant results.

## References

[1] Matsui T, Yamagishi SI, Takeuchi M, Ueda S, Fukami K, Okuda S (2010). Nifedipine inhibits advanced glycation end products (AGEs) and their receptor (RAGE) interaction-mediated proximal tubular cell injury *via* peroxisome proliferator-activated receptor-gamma activation. Biochem Biophys Res Commun.

[2] Kong LL, Wu H, Cui WP, Zhou WH, Luo P, Sun J (2013). Advances in murine models of diabetic nephropathy. J Dia Res.

[3] Habib AA, Brannagan TH (2010). Therapeutic strategies for diabetic neuropathy. Curr Neurol Neurosci Rep.

[4] Arora MK, Singh UK (2013). Molecular mechanisms in the pathogenesis of diabetic nephropathy: an update. Vasc Pharmacol.

[5] Casey RG, Joyce M, Roche-Nagle G, Chen G, Bouchier-Hayes D (2005). Pravastatin modulates early diabetic nephropathy in an experimental model of diabetic renal disease. J Surg Res.

[6] Wang RUI (2002). Two’s company, three’sa crowd: can H2S be the third endogenous gaseous transmitter?. FASEB J.

[7] Lee M, Tazzari V, Giustarini D, Rossi R, Sparatore A, Del Soldato P (2010). Effects of Hydrogen Sulfide-releasing l-DOPA Derivatives on Glial Activation potential for treating Parkinson disease. J Biol Chem.

[8] Bos EM, Leuvenink HG, Snijder PM, Kloosterhuis NJ, Hillebrands JL, Leemans JC (2009). Hydrogen sulfide-induced hypometabolism prevents renal ischemia/reperfusion injury. J Am Soc.

[9] Yamamoto J, Sato W, Kosugi T, Yamamoto T, Kimura T, Taniguchi S (2013). Distribution of hydrogen sulfide (H2S)-producing enzymes and the roles of the H2S donor sodium hydrosulfide in diabetic nephropathy. Clin Exp Nephrol.

[10] Zhao W, Zhang J, Lu Y, Wang R (2001). The vasorelaxant effect of H2S as a novel endogenous gaseous KATP channel opener. EMBO J.

[11] Zanardo RC, Brancaleone V, Distrutti E, Fiorucci S, Cirino G, Wallace JL (2006). Hydrogen sulfide is an endogenous modulator of leukocyte-mediated inflammation. FASEB J.

[12] Hu LF, Wong PTH, Moore PK, Bian JS (2007). Hydrogen sulfide attenuates lipopolysaccharide-induced inflammation by inhibition of p38 mitogen-activated protein kinase in microglia. J Neurochem.

[13] Wallace JL, Caliendo G, Santagada V, Cirino G, Fiorucci S (2007). Gastrointestinal safety and anti- inflammatory effects of a hydrogen sulfide–releasing diclofenac derivative in the rat. Gastroenterology.

[14] Li L, Rossoni G, Sparatore A, Lee LC, Del Soldato P, Moore PK (2007). Anti-inflammatory and gastrointestinal effects of a novel diclofenac derivative. Free Radic Biol Med.

[15] Trinder P (1969). Determination of blood glucose using an oxidase-peroxidase system with a non- carcinogenic chromogen. J Clin Pathol.

[16] Bonsnes RW, Taussky HH (1945). On the colorimetric determination of creatinine by the Jaffe reaction. J Biol Chem.

[17] Fawcett JK, Scott J (1960). A rapid and precise method for the determination of urea. J Clin Pathol.

[18] Grover JK, Vats V, Yadav S (2002). Effect of feeding aqueous extract of Pterocarpus marsupium on glycogen content of tissues and the key enzymes of carbohydrate metabolism. Mol Cell Biochem.

[19] Sinuani I, Averbukh Z, Gitelman I, Rapoport MJ, Sandbank J, Albeck M (2006). Mesangial cells initiate compensatory renal tubular hypertrophy *via* IL-10-induced TGF-β secretion: effect of the immunomodulator AS101 on this process. Am J Physiol Renal Physiol.

[20] Wills ED (1966). Mechanisms of lipid peroxide formation in animal tissues. Biochem J.

[21] Ellman GL (1959). Tissue sulfhydryl groups. Arch Biochem.

[22] Green LC, Wagner DA, Glogowski J, Skipper PL, Wishnok JS, Tannenbaum SR (1982). Analysis of nitrate, nitrite, and [< sup > 15</sup > N] nitrate in biological fluids. Anal Biochem.

[23] Buñag RD (1973). Validation in a wake rats of a tail-cuff method for measuring systolic pressure. J Appl Physiol.

[24] Tomohiro T, Kumai T, Sato T, Takeba Y, Kobayashi S, Kimura K (2007). Hypertension aggravates glomerular dysfunction with oxidative stress in a rat model of diabetic nephropathy. Life Sci.

[25] Haidara MA, Mikhailidis DP, Rateb MA, Ahmed ZA, Yassin HZ, Ibrahim IM (2009). Evaluation of the effect of oxidative stress and vitamin E supplementation on renal function in rats with streptozotocin-induced Type 1 diabetes. J Diabet.

[26] Gojo A, Utsunomiya K, Taniguchi K, Yokota T, Ishizawa S, Kanazawa Y (2007). The Rho-kinase inhibitor, fasudil, attenuates diabetic nephropathy in streptozotocin-induced diabetic rats. Eur J Pharmacol.

[27] Navaneethan DS, Singh S, Choudhry W (2005). Nodular glomerulosclerosis in a non-diabetic patient: Case Report and review of literature. J Nephrol.

[28] Hrabák A, Szabó A, Bajor T, Körner A (2006). Differences in the nitric oxide metabolism in streptozotocin-treated rats and children suffering from Type 1 diabetes. Life Sci.

[29] Balakumar P, Chakkarwar VA, Kumar V, Jain A, Reddy J, Singh M (2008). Experimental models for nephropathy. Journal of Renin-Angiotensin-Aldosterone System.

[30] Robertson RP (2009). β-Cell deterioration during diabetes: what’s in the gun?. Trends Endicrin Met.

[31] Mauer SM, Steffes MW, Ellis EN, Sutherland DE, Brown DM, Goetz FC (1984). Structural-functional relationships in diabetic nephropathy. J Clin Invest.

[32] Tripathi AS, Mazumder PM, Chandewar AV (2014). Changes in the pharmacokinetic of sildenafil citrate in rats with Streptozotocin-induced diabetic nephropathy. J Clin Invest.

[33] Senthil D, Choudhury GG, Mclaurin C, Kasinath BS (2003). Vascular endothelial growth factor induces protein synthesis in renal epithelial cells: A potential role in diabetic nephropathy1. Kidney Int.

[34] Almdal TP, Vilstrup H (1988). Exogenous hyperglucagonaemia in insulin controlled diabetic rats increases urea excretion and nitrogen loss from organs. Diabetologia.

[35] Murali B, Goyal RK (2001). Effect of chronic treatment with losartan on streptozotocin induced diabetic nephropathy. Clin Exp Hypertens.

[36] Piyachaturawat P, Poprasit J, Glinsukon T, Wanichanon C (1988). Gastric mucosal lesions in streptozotocin-diabetic rats. Cell Biol Int.

[37] Yamagishi SI, Nakamura K, Matsui T (2009). Regulation of advanced glycation end product (AGE)- receptor (RAGE) system by PPAR-gamma agonists and its implication in cardiovascular disease. Pharmacol Res.

[38] Maiese K (2008). Diabetic stress: new triumphs and challenges to maintain vascular longevity. Expert Rev Cardiovasc Ther.

[39] Hosseini A (2010). Benefit of magnesium-25 carrying porphyrin-fullerene nanoparticles in experimental diabetic neuropathy. Int J Nanomedicine.

[40] Fukami K, Yamagishi SI, Ueda S, Okuda S (2008). Role of AGEs in diabetic nephropathy. Curr Pharm Des.

[41] Martínez-Palacian A, Del Castillo G, Suárez-Causado A (2013). Mouse hepatic oval cells require Met-dependent PI3K to impair TGF-β-induced oxidative stress and apoptosis. PLoS One.

[42] Rodriguez WE, Sen U, Tyagi N, Kumar M, Carneal G, Aggrawal D (2007). PPAR gamma agonist normalizes glomerular filtration rate, tissue levels of homocysteine, and attenuates endothelial- myocyte uncoupling in alloxan induced diabetic mice. Int J Biol Sci.

[43] Sen U, Munjal C, Qipshidze N, Abe O, Gargoum R, Tyagi SC (2010). Hydrogen sulfide regulates homocysteine-mediated glomerulosclerosis. Am J Nephrol.

[44] Shibata R, Ueda S, Yamagishi SI, Kaida Y, Matsumoto Y, Fukami K (2009). Involvement of asymmetric dimethylarginine (ADMA) in tubulointerstitial ischaemia in the early phase of diabetic nephropathy. Nephrol Dial Transplant.

[45] Taniguchi S, Kang L, Kimura T, Niki I (2011). Hydrogen sulphide protects mouse pancreatic β‐cells from cell death induced by oxidative stress, but not by endoplasmic reticulum stress. Br J Pharmacol.

[46] Epstein PN, Overbeek PA, Means AR (1989). Calmodulin-induced early-onset diabetes in transgenic mice. Cell.

[47] Kaneko Y, Kimura T, Taniguchi S, Souma M, Kojima Y, Kimura Y (2009). Glucose-induced production of hydrogen sulfide may protect the pancreatic beta-cells from apoptotic cell death by high glucose. FEBS Lett.

[48] Samhan-Arias AK, Garcia-Bereguiain MA, Gutierrez-Merino C (2009). Hydrogen sulfide is a reversible inhibitor of the NADH oxidase activity of synaptic plasma membranes. Biochem Biophys Res Commun.

[49] Calvert JW, Jha S, Gundewar S, Elrod JW, Ramachandran A, Pattillo CB (2009). Hydrogen sulfide mediates cardioprotection through Nrf2 signaling. Circ Res.

[50] Kimura J, DaSilva K, Marshall R (2008). Population management, systems-based practice, and planned chronic illness care: integrating disease management competencies into primary care to improve composite diabetes quality measures. Dis Manag.

