# Erratum to: Combined effect of hydrogen sulphide donor and losartan in experimental diabetic nephropathy in rats

**DOI:** 10.1186/s40200-015-0212-8

**Published:** 2015-10-22

**Authors:** Manpreet Kaur, Shilpi Sachdeva, Onkar Bedi, Tavleen Kaur, Puneet Kumar

**Affiliations:** Pharmacology Division, Department of Pharmacology, ISF College of Pharmacy, Moga, 14200 Punjab India

The original version of this article [1] unfortunately contained mistakes to the figure legends and was missing Figure 7 (Fig. 1 here). The corrected legends are as follows:

**Fig. 1** Experimental Protocol design.

**Fig. 2** Effect of NaHS on blood glucose level in STZ treated rats. ^a^*P* < 0.05 versus vehicle treated, ^b^*P* < 0.05 versus [STZ (45)] treated group, ^c^*P* < 0.05 versus [STZ (45) + NaHS (10)] treated group, ^d^*P* < 0.05 versus [STZ (45) + NaHS (30)] treated group. STZ = Streptozotocin, NaHS = Sodium hydrosulphide, LOS = Losartan, DL-p = DL-propargylglycine.

**Fig. 3** Effect of NaHS and losartan on Lipid Peroxidation in STZ treated rats. ^a^*P* < 0.05 versus vehicle treated, ^b^*P* < 0.05 versus [STZ (45)] treated group, ^c^*P* < 0.05 versus [STZ (45) + NaHS (10)] treated group, ^d^*P* < 0.05 versus [STZ (45) + NaHS (30)] treated group, ^e^*P* < 0.05 versus [STZ (45) + LOS (5)] treated group. STZ = Streptozotocin, NaHS = Sodium hydrosulphide, LOS = Losartan, DL-p = DL-propargylglycine.

**Fig. 4** Effect of NaHS and losartan on Reduced Glutathione in STZ treated rats. ^a^*P* < 0.05 versus vehicle treated, ^b^*P* < 0.05 versus [STZ (45)] treated group, ^c^*P* < 0.05 versus [STZ (45) + NaHS (10)] treated group, ^d^*P* < 0.05 versus [STZ (45) + NaHS (30)] treated group, ^e^*P* < 0.05 versus [STZ (45) + LOS (5)] treated group. STZ = Streptozotocin, NaHS = Sodium hydrosulphide, LOS = Losartan, DL-p = DL-propargylglycine.

**Fig. 5** Effect of NaHS and losartan on Nitrite in STZ treated rats. ^a^*P* < 0.05 versus vehicle treated, ^b^*P* < 0.05 versus [STZ (45)] treated group, ^c^*P* < 0.05 versus [STZ (45) + NaHS (10)] treated group, ^d^*P* < 0.05 versus [STZ (45) + NaHS (30)] treated group, ^e^*P* < 0.05 versus [STZ (45) + LOS (5)] treated group. STZ = Streptozotocin, NaHS = Sodium hydrosulphide, LOS = Losartan, DL-p = DL-propargylglycine.

**Fig. 6** Effect of NaHS and losartan on MABP in STZ treated rats. ^a^*P* < 0.05 versus vehicle treated, ^b^*P* < 0.05 versus [STZ (45)] treated group, ^c^*P* < 0.05 versus [STZ (45) + NaHS (10)] treated group, ^d^*P* < 0.05 versus [STZ (45) + NaHS (30)] treated group, ^e^*P* < 0.05 versus [STZ (45) + LOS (5)] treated group. STZ = Streptozotocin, NaHS = Sodium hydrosulphide, LOS = Losartan, DL-p = DL-propargylglycine.

The missing figure can be found below, with the correct figure legend:

**Fig. 1:**
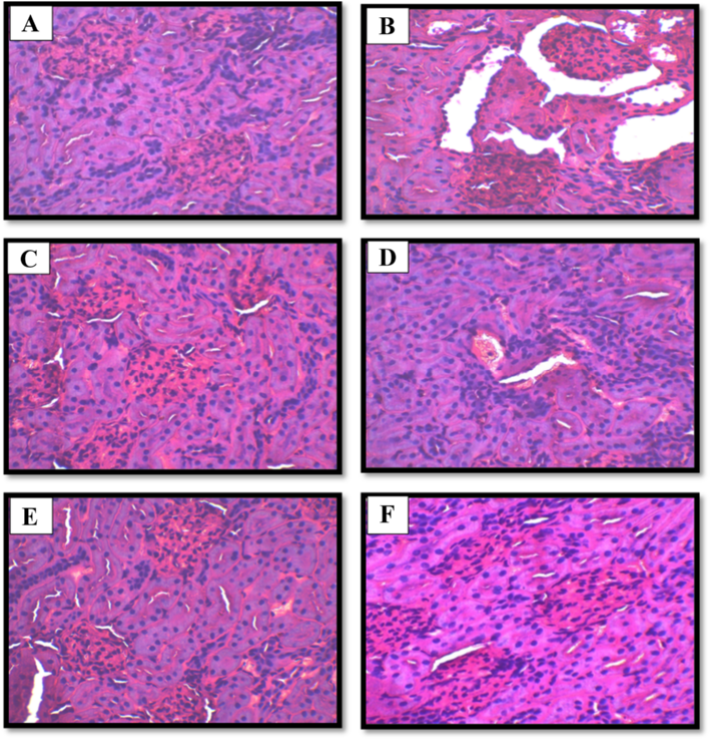
Hemotoxylin-Eosin stained longitudinal section of kidneys (10×). **a** Normal control, **b** STZ treated group (45), **c** NaHS (30), **d** DL-p (10) + NaHS (30), **e** LOS, **f** NaHS (10) + Losartan (5)
